# Ginsenoside Rg3 Decreases Fibrotic and Invasive Nature of Endometriosis by Modulating miRNA-27b: *In Vitro* and *In Vivo* Studies

**DOI:** 10.1038/s41598-017-17956-0

**Published:** 2017-12-15

**Authors:** Min Kyoung Kim, Seung Kyun Lee, Ji Hyun Park, Jae Hoon Lee, Bo Hyon Yun, Joo Hyun Park, Seok Kyo Seo, SiHyun Cho, Young Sik Choi

**Affiliations:** 1 Department of Obstetrics and Gynecology, Severance Hospital, Yonsei University College of Medicine, Seoul, 03722 Republic of Korea; 2 Institute of Women’s Life Medical Science, Yonsei University College of Medicine, Seoul, 03722 Republic of Korea; 3 Department of Obstetrics and Gynecology, Fertility Center of CHA Gangnam Medical Center, CHA University, Seoul, 06125 Republic of Korea; 4 Department of Obstetrics and Gynecology, Gangnam Severance Hospital, Yonsei University College of Medicine, Seoul, 06273 Republic of Korea

## Abstract

This research aimed to evaluate the potential therapeutic effects of Rg3 on endometriosis and identify target miRNAs. We designed an *in vitro* study using human endometrial stromal cells (HESCs) obtained from patients with endometriosis and an *in vivo* study using mouse models. HESCs were treated with Rg3-enhanced red ginseng extract (Rg3E); real-time PCR and microarray profiling, transfection, and western blot were performed. Mouse endometriosis models were developed and supplemented with Rg3E for 8 weeks. Gross lesion size and fibrotic character were analyzed in the mouse models. RNA levels of Ki-67, col-1, CTGF, fibronectin, TGF-β1, MMP2 and MMP9 significantly decreased in HESCs after Rg3E treatment. Microarray analysis revealed downregulation of miR-27b-3p, which is related to fibrosis modulation. Expression of miR-27b-3p was significantly higher in HESCs from patients with endometriosis than that of controls, and Rg3E treatment significantly decreased its expression; the contraction and migration assay revealed significant reductions in both fibrosis and migration potential in Rg3E-treated HESCs from endometriosis patients. A decrease in size and fibrotic character of endometrial lesions from the Rg3E groups was observed *in vivo*. In conclusion, Rg3 effectively altered fibrotic properties of HESCs from patients with endometriosis, which is likely associated with miR-27b-3p modulation.

## Introduction

Endometriosis is a common benign gynecological disorder, affecting up to 10% of all women of reproductive age and 20–50% of women with chronic pelvic pain and/or infertility^1^. It is defined as the proliferation of endometrial tissue outside of the uterine cavity^2^. The exact pathogenic mechanisms of endometriosis are not known and diagnosis is often delayed—making it more difficult to treat, both medically and surgically. However, several studies demonstrate that endometriosis has an invasive and fibrotic nature that is induced by inflammation^3,4^, which may represent a key pathophysiological target for treatment.

MicroRNAs (miRNAs) are a family of endogenous, small, noncoding, functional RNAs that control gene expression by translational repression or degradation of messenger RNA transcripts after targeting the 3′-untranslated region (3′-UTR)^5^. Several studies have demonstrated that miRNAs are important regulators of development and cellular homeostasis through their control of diverse biological processes. Aberrant miRNA expression is known to be associated with various human diseases such as cancer, cardiovascular and inflammatory disorders, as well as gynecological diseases^6–10^. Previously, we showed that several circulating miRNAs such as let-7b, 7d, and 7 f are differentially expressed in the sera of endometriosis patients^5^. Several other studies also identified differential expression of miRNAs in eutopic and ectopic endometrial samples from endometriosis patients using microarray profiling^11–13^. It has been suggested that miRNAs act as potent regulators of gene expression in the pathogenesis of endometriosis and its associated reproductive disorders^14^.

Korean red ginseng (KRG) has been traditionally used as an herbal medicine to treat various diseases in Eastern Asia. Recent studies have shown that KRG has various biological activities such as immune enhancement, antioxidant, anti-inflammatory, neuroprotective, anti-metabolic syndrome, and anti-menopausal disorder effects^15–19^. Ginsenosides are biologically active components of KRG, and there are many different types of ginsenosides with different pharmacological activities^20^. Ginsenoside Rg3 is a major active component of KRG and has been shown to have various pharmacological benefits such as immunomodulatory, antioxidant, anti-inflammatory, anticancer, and anti-aging activities in several diseases and infections^21,22^. Previous studies demonstrated that Rg3-enhanced red ginseng extract (Rg3E) has anti-inflammatory effects in asthmatic lung tissue, brain, hepatic and renal injury^23–25^. Also, many types of ginsenosides were studied to be associated with fibrosis. Ginsenoside Rh2 improved cardiac fibrosis by increasing PPAR*δ* signaling^26^. Ginsenoside Rg1 was shown to reduce cigarette smoke-induced airway fibrosis by inhibiting TGF-*β*/Smad pathway in a rat model^27^. Rg3 was also revealed to be anti-fibrotic in hepatic fibrosis of mouse model^28^. Considering the anti-inflammatory and anti-fibrotic activity of ginsenosides, we hypothesized that Rg3E effectively inhibits the key pathophysiology of endometriosis.

Matsuzaki *et al*. described the fibrosis in endometriosis is induced by the Wnt/*β*-catenin signaling pathway^29^. Treating the endometrial stromal cells of endometriosis patients with antagonists of Tcf/*β*-catenin complexes significantly decreased the expression of fibrotic markers such as *α*-smooth muscle actin, type I collagen (Col-1), connective tissue growth factor (CTGF), and fibronectin. They also revealed that inhibiting aberrant activation of Wnt/*β*-catenin signaling also results in hindering cell proliferation, migration, and/or invasion of endometrial stromal cells of patients with endometriosis^30^. Therefore, we hypothesized that Rg3E may also have the potential to inhibit the Wnt/*β*-catenin signaling pathway to decrease the cellular fibrosis, proliferation, and/or invasion of endometrial stromal cells.

The objective of this study was to investigate whether Rg3E affects the key pathological processes of endometriosis such as invasion, proliferation, and fibrosis *in vitro* and *in vivo*. Additionally, we analyzed the role of miRNAs in a pathogenic condition previously described^31^ and evaluated the therapeutic effects of Rg3E by targeting specific miRNAs found from the analysis.

## Results

### Patient Characteristics

There were no significant differences in age and body mass index (BMI) between the endometriosis group and controls. However, the endometriosis group had significantly lower gravidity (0.64 ± 0.34 vs. 2.18 ± 0.50, *P* = 0.019) and parity (0.45 ± 0.25 vs. 1.36 ± 0.28, *P* = 0.024) than those of the controls, and serum CA-125 levels were significantly higher than those of the controls (69.18 ± 17.56 vs. 15.14 ± 2.90, *P* = 0.015) (Table 1).

**Table 1:** Patient characteristics.

	Control (N = 15)	Endometriosis (N = 21)	*P*-value
Age (years)	36.09 ± 1.97	33.91 ± 2.33	0.482
Gravida	2.18 ± 0.50	0.64 ± 0.34	0.019
Parity	1.36 ± 0.28	0.45 ± 0.25	0.024
BMI (kg/m^2^)	22.16 ± 0.74	20.27 ± 0.63	0.065
CA125 (IU/ml)	15.14 ± 2.90	69.18 ± 17.56	0.015

^#^The data were expressed in mean ± SEM.

### Effects of Rg3E on Cell Proliferation, Invasion, Apoptosis, and Fibrosis Markers

**Figure 1:**
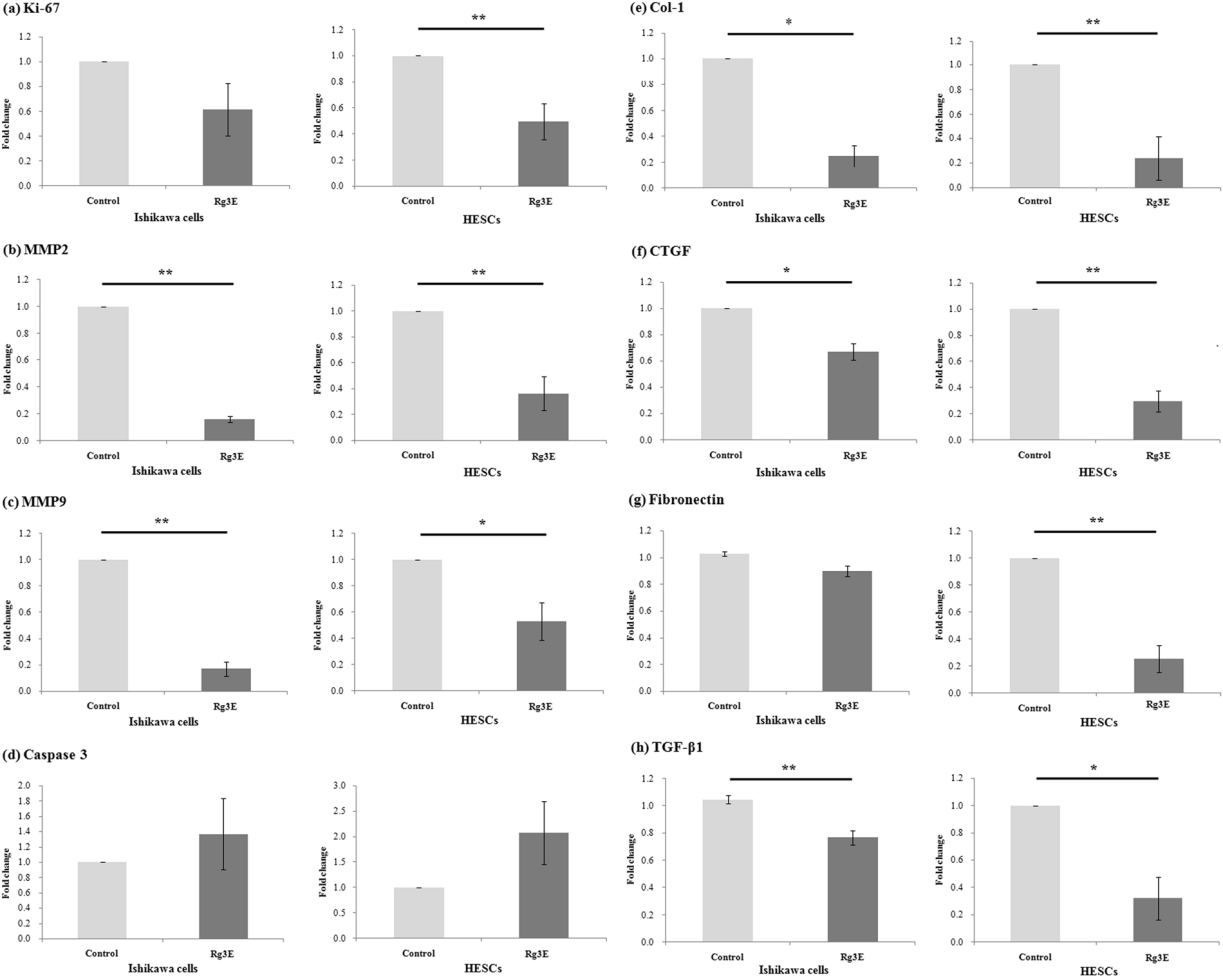
mRNA concentrations of Ki-67, MMP2, MMP9, Caspase 3 and fibrosis markers after Rg3E treatment in Ishikawa cells and HESCs in patients with endometriosis. Both types of cells were treated with Rg3E for 48 h before analysis. Quantitative real-time polymerase chain reaction (qRT-PCR) amplification was performed using the 7300 Real Time PCR System (Applied Biosystems, Foster City, CA, USA). The mRNA concentrations of each sample were normalized to GAPDH expression. (**a**) Ki-67 was significantly decreased in HESCs after Rg3E treatment. Decreasing trend was noted in Ishikawa cells without statistical significance. (**b**) MMP2 and (**c**) MMP9 were significantly downregulated in both Ishikawa cells and HESCs. (**d**) Caspase 3 showed increasing trend without statistical significance in both Ishikawa cells and HESCs. The fibrosis markers, (**e**) Col-1 (**f**) CTGF (**g**) fibronectin and (**h**) TGF-*β*1 were all significantly decreased in HESCs. (*P < 0.05. **P < 0.01. N = 8).

The MTT assay, used to test the toxicity of Rg3E at concentrations of 0, 400, 800, 1200, and 1600 µg/mL, showed significant decreases in cell viability at 800 µg/mL. Therefore, 400 µg/mL Rg3E was used for all subsequent experiments (Supplementary Information). Ki67, a cell proliferation marker, showed significantly decreased level in Rg3E-treated human endometrial stromal cells (HESCs) (0.5 fold decrease, *P* = 0.005); however, no significant changes were observed in Ishikawa cells (0.62 fold decrease, *P* = 0.213). Levels of matrix metalloproteinase (MMP)-2 and MMP9, markers of invasion, significantly decreased in Rg3E-treated HESCs (1.0 vs. 0.36, *P* = 0.002 in MMP2; 1.0 vs. 0.53, *P* = 0.014 in MMP9) and Ishikawa cells (0.16 fold decrease, *P* = 0.001 in MMP2; 0.17 fold decrease, *P* = 0.004 in MMP9). In contrast, caspase 3, a marker of apoptosis, was not significantly changed in either the Ishikawa cells (1.37 fold increase, *P* = 0.514) or HESCs (2.07 fold increase, *P* = 0.118) after Rg3E treatment. Markers of fibrosis, including type 1 collagen (Col-1) (0.24 fold decrease, *P* = 0.002 in HESCs; 0.25 fold decrease, *P* = 0.012 in Ishikawa cells), connective tissue growth factor (CTGF) (0.3 fold decrease, *P* < 0.001 in HESCs; 0.67 fold decrease, *P* = 0.031 in Ishikawa cells), fibronectin (0.25 fold decrease, *P* = 0.002), and transforming growth factor (TGF)-*β*1 (0.32 fold decrease, *P* = 0.013) showed significantly decreased levels after treatment with Rg3E in HESCs (Fig. 1).

### Effects of Rg3E on Cell Migration and Collagen Gel Contraction

**Figure 2:**
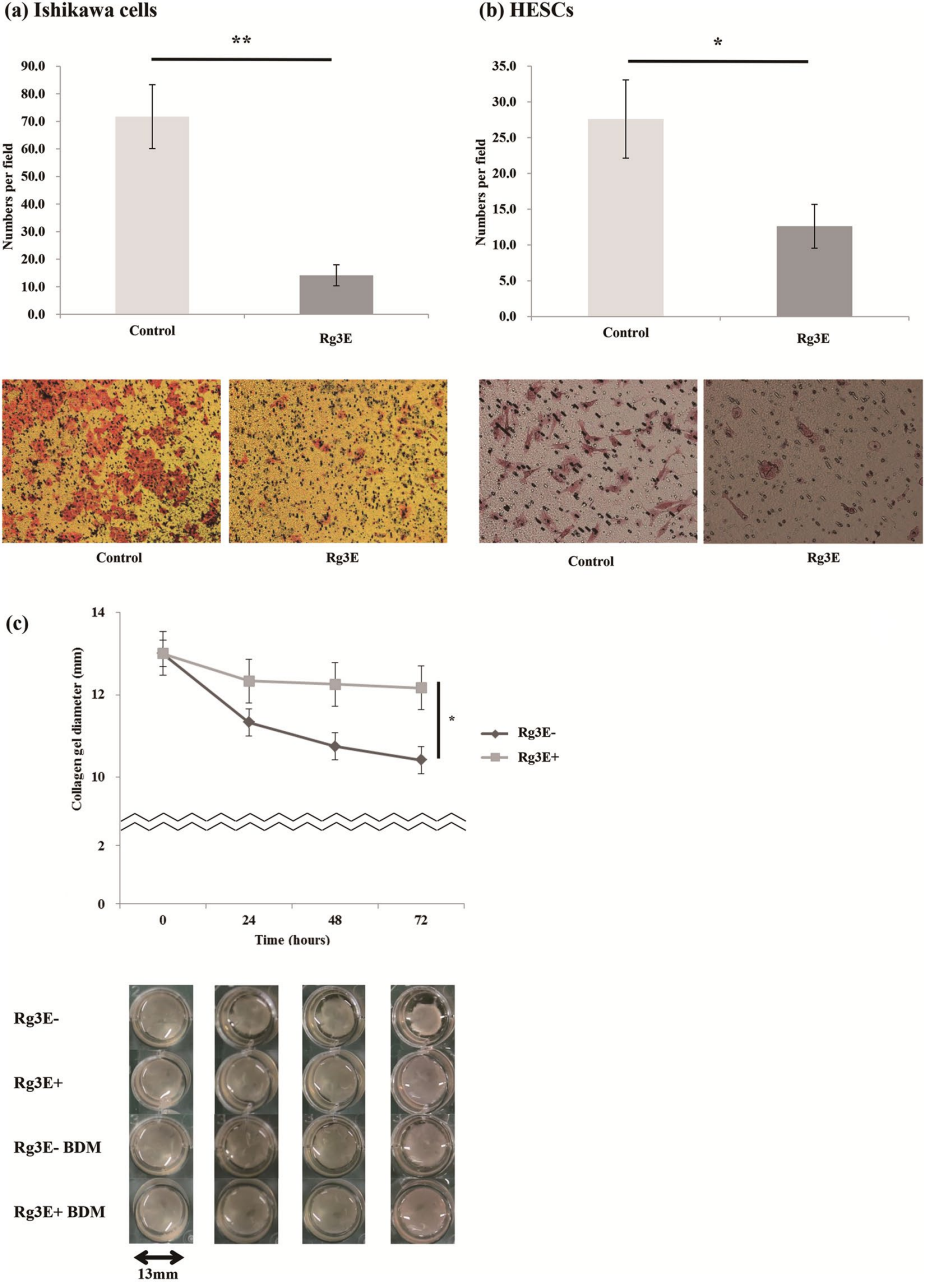
Migration assay of (**a**) Ishikawa cells and (**b**) HESCs of the patients with endometriosis after 48h-Rg3E treatment and (**c**) Contraction gel assays of HESCs of the patients with endometriosis before and after 72 hours of Rg3E treatment. (*P < 0.05. **P < 0.01). (**a**,**b**) Migration assays in Ishikawa cells (cell count: 71.75 vs. 14.15, *P* = 0.003, N = 7) and HESCs (cell count: 27.61 vs. 12.62, *P* = 0.026, N = 12) of patients with endometriosis showed significantly decreased cell count. (**c**) Collagen gel contraction assay after 72 hours of Rg3E treatment revealed significantly less collagen gel contraction compared to the control group. (Collagen gel diameter (mm): 12.17 vs. 10.42, *P* = 0.012, N = 6) (Rg3E−: without Rg3E treatment; Rg3E+: with Rg3E treatment; BDM: 3-Butandione manaxime treatment).

The migration assay showed a significant decrease in cell number after Rg3E treatment for both Ishikawa cells (cell count: 71.75 vs. 14.15, *P* = 0.003, N = 7) and HESCs (cell count: 27.61 vs. 12.62, *P* = 0.026, N = 12) (Fig. 2(a,b)). Collagen gel contractility of HESCs was evaluated using the collagen gel contraction assay. After treatment with Rg3E for 72 h, collagen gel contraction was significantly less than that of the control group that did not receive Rg3E treatment (contraction gel diameter: 10.42 vs. 12.17, *P* = 0.012, N = 6) (Fig. 2(c)).

### miRNA Profiling after Rg3E Treatment and Validation of miR-27b-3p in Endometriosis

**Figure 3:**
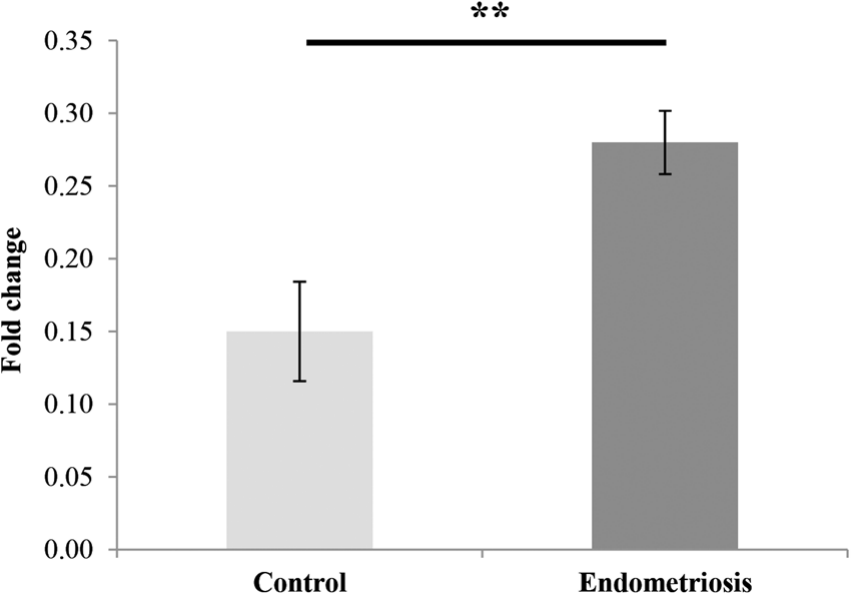
Expressions of miR-27b-3p in eutopic endometrium of the patients with and without endometriosis. The Affymetrix GeneChip^®^ miRNA array process was conducted per the manufacturer’s protocol. A comparative analysis between test and control samples was carried out using fold-change. The basal miR-27b-3p expression was significantly increased in endometriosis patients compared to control. (*P < 0.05. **P < 0.01. N = 10).

**Figure 4:**
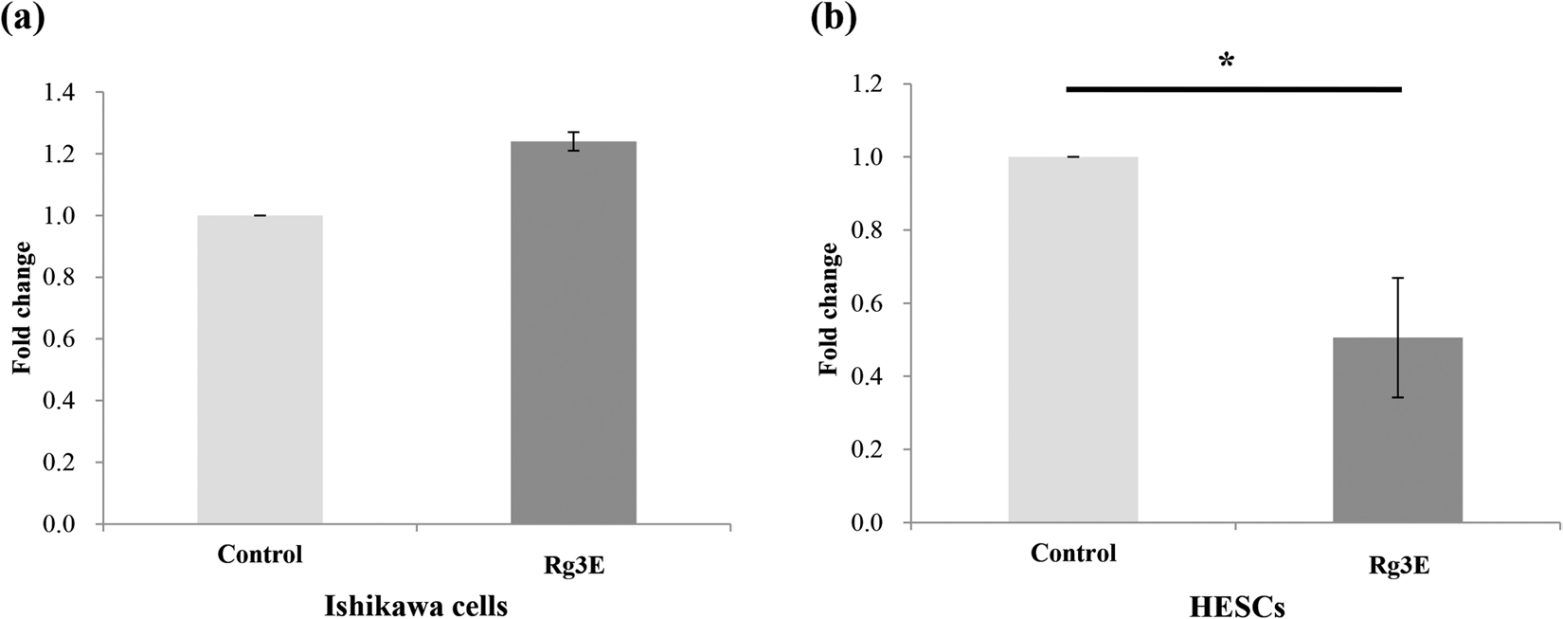
Expressions of miR-27b-3p in **(a)** Ishikawa cell lines and **(b)** HESCs from the patients with endometriosis after Rg3E treatment. Both types of cells were treated with Rg3E for 48 h before microarray analysis. The Affymetrix GeneChip^®^ miRNA array process was conducted per the manufacturer’s protocol. A comparative analysis between test and control samples was carried out using fold-change. The miR-27b-3p expression was significantly downregulated in endometriosis patient’s HESCs after Rg3E treatment. MiR-27b-3p is known for its correlation with fibrosis and its downregulation by Rg3E demonstrates the possibility of Rg3E decreasing fibrotic nature of endometriosis. (*P < 0.05. N = 6).

To evaluate the effects of Rg3E on miRNAs associated with endometriosis, miRNA profiling was performed after Rg3E treatment of HESCs from patients with endometriosis. MicroRNA microarray analysis of samples from patients with endometriosis revealed several upregulated miRNAs. After Rg3E treatment, 20 miRNAs were significantly upregulated more than two-fold and six miRNAs were significantly downregulated more than two-fold; miR-27b-3p was one of the most downregulated miRNAs after Rg3E treatment in HESCs from patients with endometriosis. The miR-27b-3p is specifically related to fibrosis and is an important characteristic of endometriosis. To validate the miRNA microarray results, expression levels of miR-27b-3p were compared between eutopic endometria from patients with endometriosis and those without the disease. In the eutopic endometrium of patients with endometriosis, miR-27b-3p expression was approximately two-fold higher than that observed in patients without the disease (fold change 0.28 vs. 0.15, *P* = 0.004) (Fig. 3). After Rg3E treatment, miR-27b-3p expression significantly decreased in HESCs (0.51 fold decrease, *P* = 0.029) (Fig. 4(b), Table 2); however, similar changes were not observed in Ishikawa cells.

**Table 2:** Microarray analysis and significant fold changes of miRNAs before and after 48h-Rg3E treatment in HESCs of patients with endometriosis.

**Up-regulated**			**Down-regulated**		
**miRNA**	**FC**	***P*** **-value**	**miRNA**	**FC**	***P*** **-value**
hsa-miR-3188	3.121276	0.030	hsa-miR-22-5p	−7.511362	0.011
hsa-miR-4674	3.102003	0.049	hsa-miR-154-5p	−3.965245	0.009
hsa-miR-6805-5p	2.813756	0.026	hsa-miR-654-3p	−2.518972	0.041
hsa-miR-4516	2.734496	0.030	**hsa-miR-27b-3p**	−**2.454641**	**0.029**
hsa-miR-6765-5p	2.675679	0.041	hsa-miR-28-3p	−2.216515	0.049
hsa-miR-8089	2.599531	0.046	hsa-miR-140-5p	−2.207144	0.033
hsa-miR-6821-5p	2.59466	0.044			
hsa-miR-3621	2.545324	0.034			
hsa-miR-4649-5p	2.539717	0.0001			
hsa-miR-1908-5p	2.488741	0.048			
hsa-miR-4486	2.44996	0.033			
hsa-miR-8069	2.369964	0.034			
hsa-miR-663a	2.33128	0.035			
hsa-miR-6781-5p	2.241129	0.026			
hsa-miR-4690-5p	2.17942	0.035			
hsa-miR-6729-5p	2.146922	0.029			
hsa-miR-149-3p	2.140209	0.027			
hsa-miR-6125	2.111751	0.031			
hsa-miR-4745-5p	2.092432	0.047			
hsa-miR-937-5p	2.086316	0.038			

There were six significantly decreased miRNAs and miR-27b-3p (previously reported to be associated with modulating fibrosis) was included. Functions of other decreased miRNAs were yet to be discovered or unrelated to the major pathogenic characteristics of endometriosis. Additionally, there were twenty miRNAs that were significantly increased after Rg3E treatment. However, these miRNAs’ roles were not yet clarified.

^#^FC: fold change. FC > 2 and *P* < 0.05 were considered significant.

### miR-27b-3p Inhibitor Transfection and Western Blot

**Figure 5:**
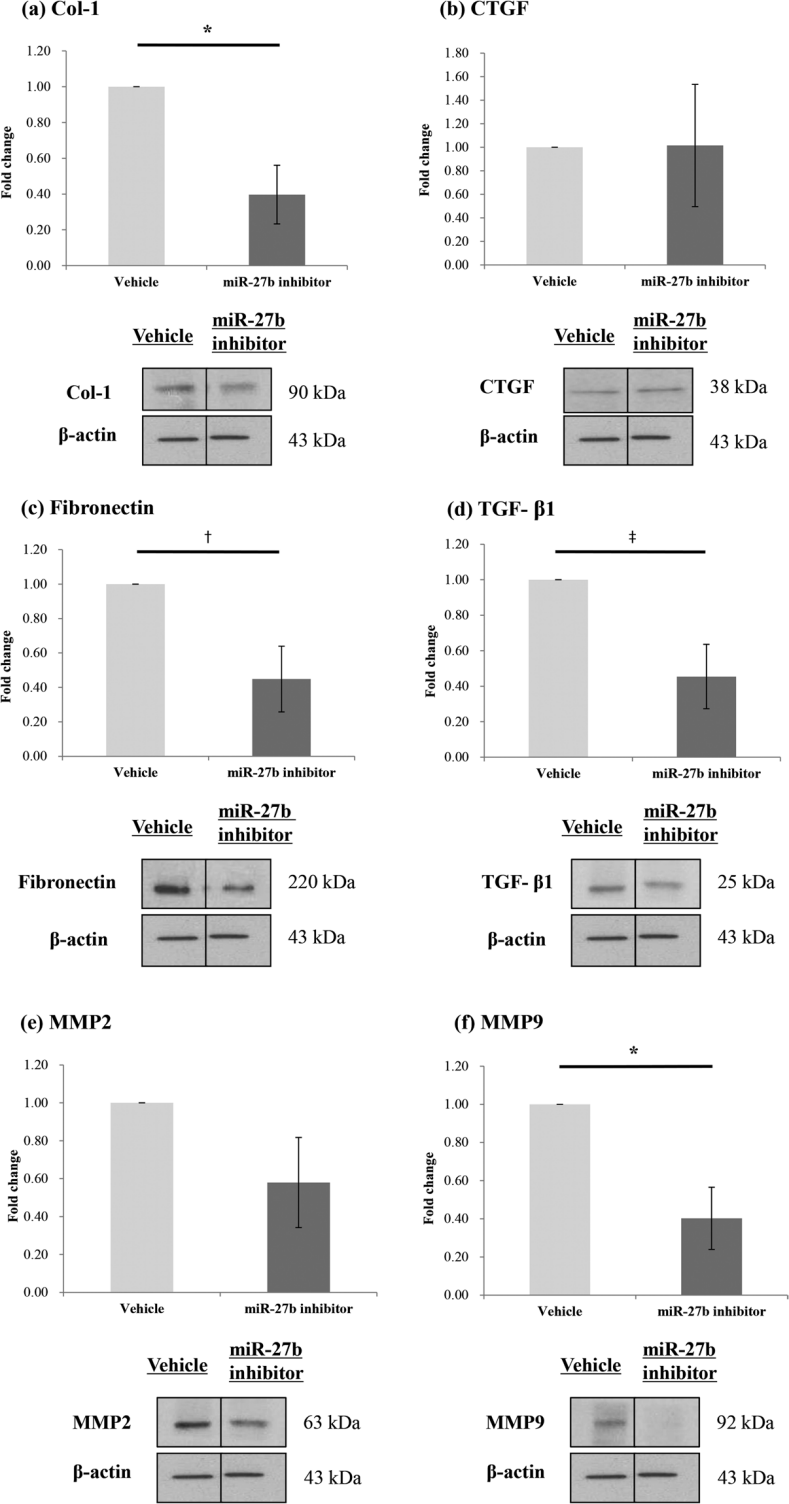
mRNA concentrations and protein expressions of Col-1, CTGF, Fibronectin, TGF-*β*1, MMP2 and MMP9 in HESCs from the patients with endometriosis after mir-27b-3p inhibitor transfection. Cells were transfected with miR-27b-3p inhibitor for 48 h, and then subjected to qRT-PCR and western blot analysis to determine the mRNA concentrations and protein expression levels of Col-1, CTGF, Fibronectin, TGF-*β*1, MMP2 and MMP9. The mRNA concentrations and protein expressions of (**a**) Col-1 was significantly decreased. (**b**) CTGF did not show significant difference after Rg3E treatment. Although not statistically significant, (**c**) fibronectin and (**d**) TGF-*β*1 showed decreasing trend after the treatment. (**e**) MMP2 did not show significant difference but (**f**) MMP 9 was significantly decreased. (*P < 0.05. ^†^ *P* = 0.063. ^‡^ *P* = 0.057. N = 4).

To evaluate transfection efficacy, miR-27b-3p expression was measured 48 h after transfection with an hsa-miR-negative control and hsa-miR-27b-3p inhibitor in HESCs from the endometrium of the patients with endometriosis. Expression of miR-27b-3p was 100- to 200-fold lower after treatment with the hsa-miR-27b-3p inhibitor than that observed after treatment with the hsa-miR-negative control (Supplementary data). Transfection of the miR-27b-3p inhibitor downregulated markers of fibrosis in HESCs (Fig. 5). Col-1 mRNA concentration and protein expression significantly decreased after treatment with the miR-27b-3p inhibitor (0.40 fold decrease, *P* = 0.035), whereas that of CTGF (1.02 fold increase, *P* = 0.979), fibronectin (0.45 fold decrease, *P* = 0.063), and TGF-*β*1 (0.45 fold decrease, *P* = 0.057) did not significantly change. MMP2 and MMP9, markers of invasion, were also affected by transfection of the miR-27b-3p inhibitor. Both mRNA concentration and protein expression of MMP9 were significantly downregulated (0.40 fold decrease, *P* = 0.035), and those of MMP2 also decreased; however, the results for MMP2 were not significant.

### Mouse Model of Endometriosis

**Figure 6:**
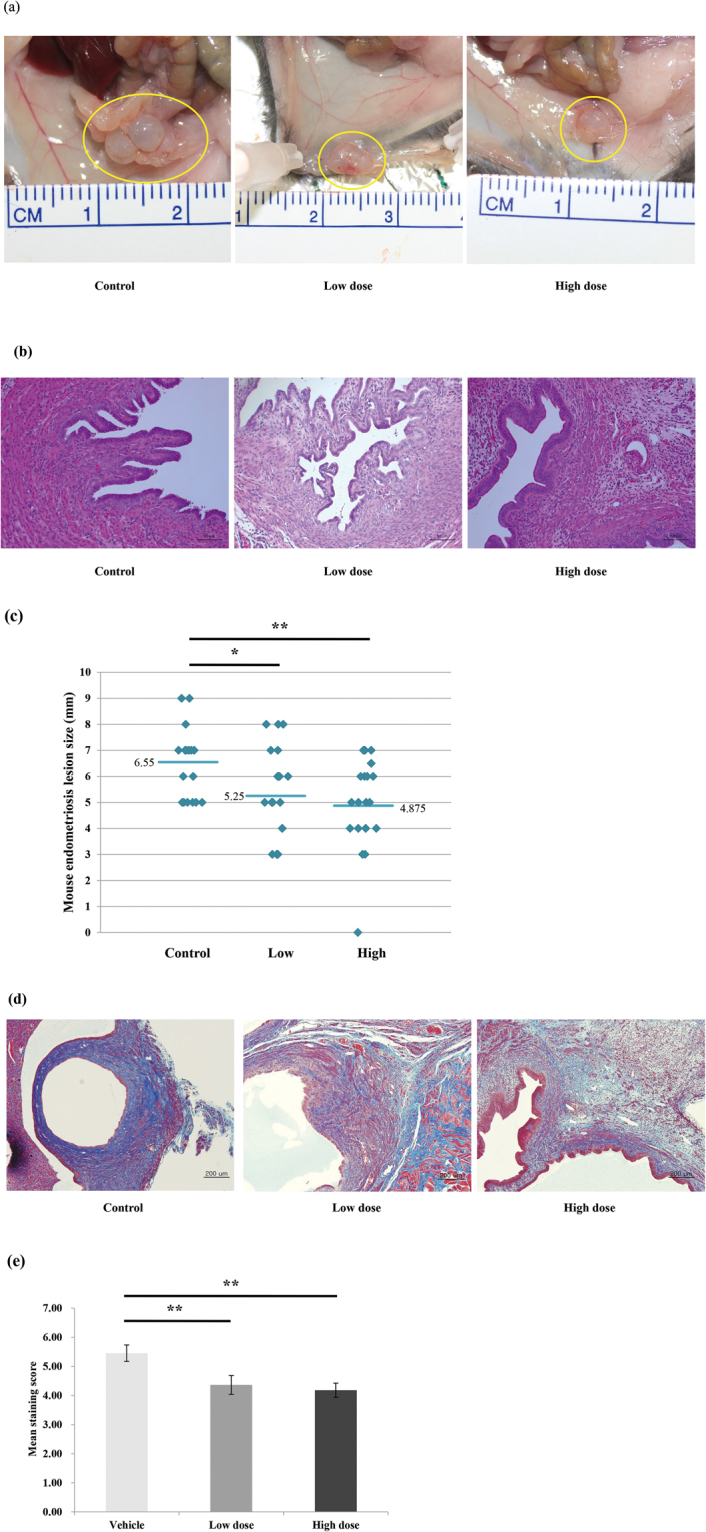
Mouse endometriosis model results. (**a**) Photographs showing endometriotic implants in mouse model fed with vehicle, low dose (0.1 mg/g), and high dose (0.2 mg/g) of Rg3E for 8 weeks. (**b**) H&E staining of the endometriotic implants of each treatment groups (x200 magnification) (**c**) Comparisons of the size of the endometriotic implants according to different treatment dose shows significantly decreasing size as the treatment dose increases. (**d**) Masson’s trichrome staining of endometriotic implants according to different treatment dose. (x100 magnification) (**e**) Comparisons of Masson’s trichrome staining scores according to different treatment dose also shows significant decreases as the treatment dose increases. (**P < 0.01. N = 10 per group).

After 8 weeks of Rg3E treatment, the mice were sacrificed and their endometriotic lesions were obtained. All transplanted endometriotic lesions were found in the peritoneum of the sacrificed mice. The mean diameter of lesions in the vehicle, low-dose, and high-dose groups was 6.55 mm, 5.25 mm, and 4.88 mm, respectively. The average implant size in each group decreased as the dose of Rg3E was increased (Fig. 6(a–c)). Lesions from the treatment groups were significantly smaller than those from the vehicle group (vehicle vs. low dose, *P* = 0.007, vehicle vs. high dose, *P* = 0.006, N = 10 per group). However, no significant differences were noted between treatment groups.

Endometriotic lesions were stained with Masson’s trichrome stain, and mean staining scores were calculated. Similar to the results observed for lesion size, mean staining scores for implants from each group decreased as the dose of Rg3E was increased. Mean staining scores of the vehicle, low-dose, and high-dose groups were 5.45, 4.36, and 4.18, respectively. Treatment group scores were significantly smaller than those of the vehicle group (vehicle vs. low dose, *P* = 0.006, vehicle vs. high dose, *P* = 0.011) (Fig. 6(d,e)).

**Figure 7:**
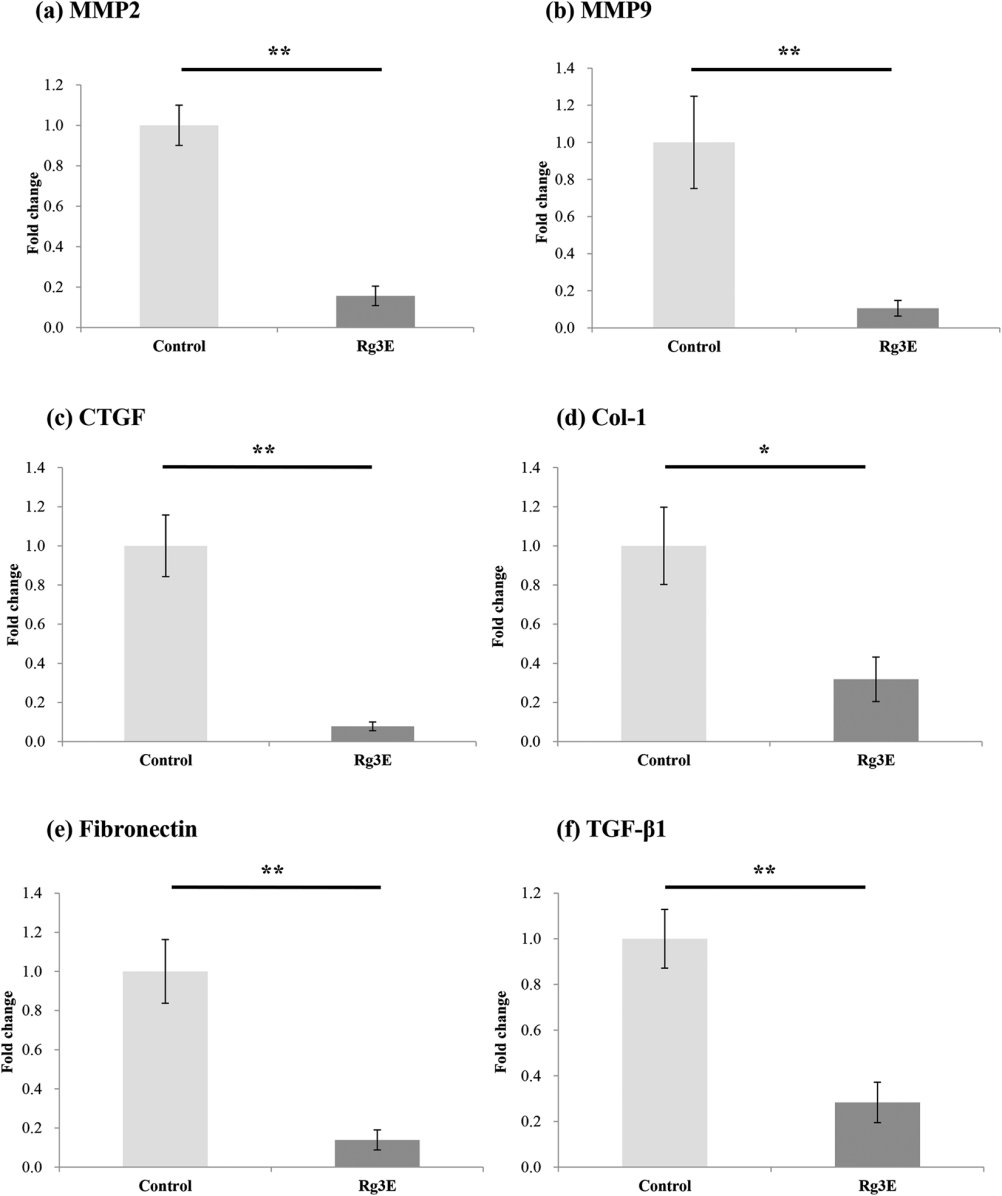
*In vivo* mRNA concentrations of MMP2, MMP9, CTGF, Col-1, fibronectin, and TGF-*β*1 after Rg3E treatment for 8 weeks. Quantitative RT-PCR amplification was performed using the 7300 Real Time PCR System (Applied Biosystems, Foster City, CA, USA). The mRNA concentrations of each sample were normalized to GAPDH expression. The invasion markers, (**a**) MMP2 and (**b**) MMP9, and fibrosis markers, (**c**) CTGF (**d**) Col-1 (**e**) fibronectin and (**f**) TGF-*β*1, all decreased significantly for mouse with 8-weeks of Rg3E treatment compared to control mice. (*P < 0.05. **P < 0.01. N = 5).

*In vivo* mRNA concentrations of the invasion and fibrosis markers were examined by qRT-PCR. Similar to the *in vitro* study results, MMP2, MMP9, CTGF, Col-1, fibronectin and TGF-*β*1 were all significantly downregulated in mice with 8-weeks of Rg3E treatment compared to control mice (Fig. 7).

## Discussion

This study demonstrates that Rg3E from KRG significantly alters several major pathogenic characteristics of endometriosis, both *in vitro* and *in vivo*. To our knowledge, this report is the first study to evaluate the effects of Rg3E on endometriosis. The treatment mechanism involved changes in several miRNAs, including miR-27b-3p. In this study, we show that miR-27b-3p expression is elevated in the eutopic endometrium of patients with endometriosis, and Rg3E effectively reduces expression of this miRNA in HESCs from patients with endometriosis. Modulation of miR-27b-3p is associated with alteration of several cellular characteristics of endometriosis, including cell proliferation and invasion. Among these changes, the most profound effect was seen in fibrosis formation. We showed that Rg3E and miR-27b-3p inhibition effectively reduce endometriosis fibrotic potential using a contraction gel assay and an *in vivo* mouse model.

Endometriosis is considered a benign disease; however, it often presents characteristics of malignancy such as proliferation and invasion^32^, and previous studies have shown that Rg3 inhibits such characteristics. A Chinese study demonstrated that Rg3 stimulates apoptosis and exhibits antitumor activity against lung cancer cells *in vitro* ^22^. Rg3 is also known to suppress pro-angiogenic (TNF-α) and immunosuppressive cytokine (TGF-β) secretion, which may promote Rg3-induced immunogenic tumor cell death^33^. The invasive properties of endometriosis are related to an increase in proteolytic activity and matrix remodeling. MMPs are important for degrading the extracellular matrix, which takes part in endometriosis development^34^. This study shows that Rg3E significantly downregulates MMP2 and MMP9, blocking a significant pathogenic pathway of endometriosis.

The most interesting point of this study is inhibition of fibrosis by Rg3E. Fibrosis is also an important pathogenic characteristic of endometriosis that aggravates infertility and pelvic pain. A previous study demonstrated that the Wnt/*β*-catenin signaling pathway is involved in regulating the cellular and molecular mechanisms of fibrosis in endometriosis, and the Tcf/*β*-catenin complex decreases fibrotic markers^29^. Another recent study claimed that endometriotic mesenchymal stem cells significantly promote fibrogenesis in ovarian endometrioma by paracrine production of TGF-*β*1 and Wnt1^35^. A study using TGF- *β*1 knock-out mice also has shown that TGF- *β*1 deficiency suppresses endometriotic lesion development, which emphasizes the role of TGF- *β*1 in endometriosis^36^. The present study shows Rg3E’s anti-fibrotic effects in HESC-culture experiments and a collagen gel contraction assay, as well as fibrosis-related miRNA transfection. Previously, only one animal study reported the relationship between Rg3 and fibrosis, showing that Rg3 inhibits hepatic fibrosis in murine *Schistosomiasis japonica* models^28^. In this study, Rg3E greatly decreased expression of all the fibrosis-related markers that we analyzed (CTGF, fibronectin, Col-1, and TGF-*β*). Results differed among the control and Rg3E-treated groups, as shown by *in vitro* experiments and *in vivo* through Masson’s trichrome staining of mouse endometrial lesions. Although we discovered the miRNA related to this phenomenon, further studies will be needed to confirm the specific mechanism of Rg3′s anti-fibrotic activity.

Recently, several studies have described the relationship between miRNAs and endometriosis pathogenesis. A recent study revealed that lower levels of miR-200b, miR-15a-5p, miR-19b-1-5p, miR146a-5p, and miR-200c, and higher levels of miR-16-5p, miR-106b-5p, and miR-145-5p are related to modulation of vascular endothelial growth factor A (VEGFA), epidermal growth factor receptor 2 (EGFR2), phosphatase and tensin homolog (PTEN), and C-X-C chemokine receptor type 4 (CXCR4) expression, which are important in the pathogenesis of endometriosis^37^. Another study showed that miR-503, a miRNA that is repressed in endometriosis, induces apoptosis and inhibits cell proliferation, angiogenesis, and contractility of human ovarian endometriotic stromal cells^38^.

Among the miRNAs examined in this study, miR-27b was particularly highly expressed in the endometrium of patients with endometriosis. Several previous studies suggest that miR-27b is involved in fibrosis development. Overexpression of miR-27b promotes hypertrophic cardiomyocyte growth, while its suppression leads to inhibition of hypertrophic cell growth^39^. miR-27b expression significantly increased in both the sclerotic intima and serum samples of arteriosclerosis obliterans patients^40^. In pulmonary fibrosis, overexpression of miR-27b increased the expression of alpha smooth muscle actin (α-SMA)^41^. All these studies have shown that miR-27b expression is induced by TGF-*β*1, which is also related to fibrosis. Although there were some discrepancies in results from PCR and transfection in this study, we speculate that Rg3 affects various types of miRNAs simultaneously to inhibit fibrosis. The transfection experiment was specifically proceeded with miR-27b-3p inhibitor that other fibrosis markers which were not significantly changed may be regulated mainly by other types of miRNAs. Therefore, increased expression of miR-27b in endometriosis may be related to its fibrotic characteristics. More importantly, decreased miR-27b by Rg3E treatment demonstrates that Rg3 may be effective for reducing the fibrotic nature of the disease.

In conclusion, Rg3 is shown to have beneficial effects for reducing the proliferative, invasive, and fibrotic nature of endometriosis. Among the endometriosis-affected miRNAs, miR-27b-3p was especially related to the development of fibrosis and inhibition of miR-27b significantly reduced fibrosis. In addition, Rg3 downregulated miR-27b-3p expression in HESCs. Therefore, Rg3 and modulation of associated miRNAs may be an adjuvant therapy for endometriosis.

## Subjects and Methods

### Study Population and Sample Collection

Thirty-six Korean female patients aged from 21–49 (premenopausal women) were enrolled in this study, and written informed consent was obtained before surgery in accordance with the study protocol, which was approved by the Institutional Review Board of Gangnam Severance Hospital. Patients included in this study underwent laparoscopy for endometriosis or other benign ovarian cysts. Twenty-one patients were histologically confirmed to have endometriosis. Fifteen patients with histologically confirmed other benign ovarian cysts were included as controls. Postmenopausal women, previous hormone or GnRH agonist users, and patients who had adenomyosis, endometrial cancer, endometrial hyperplasia or endometrial polyps, infectious diseases, chronic or acute inflammatory diseases, malignancy, autoimmune diseases, or cardiovascular diseases were excluded.

### Culture of Primary Endometrial Stromal Cells and Ishikawa Cell Line

Eutopic endometrial tissue was obtained from 21 patients with endometriosis and 15 patients with benign ovarian cysts by endometrial biopsy before surgical procedures. Most of the endometrial tissue samples were obtained at secretory phase and very few were at proliferative phase. The phase of the cycle was confirmed by calculating the menstrual cycle date. The tissue samples were minced into smaller pieces and incubated in Hank’s balanced salt solution including HEPES (25 mmol/mL), 1% penicillin/streptomycin, collagenase (1 mg/mL, 15 U/mg), and deoxyribonuclease (0.1 mg/mL, 1,500 U/mg) for 60 min at 37 °C with agitation and pipetting. The cells were pelleted, washed, suspended in Ham F12:DMEM (1:1) containing 10% fetal bovine serum (FBS) and 1% penicillin/streptomycin and then passed through a 40-µm cell strainer (Falcon) and plated into 75 cm^2^ Falcon tissue culture flasks (BD Biosciences, San Jose, CA, USA). Cultured HESCs at 3–5 passages were used for further analysis. Ishikawa cells were maintained in MEM (Invitrogen, Carlsbad, CA, USA) containing 2.0 mmol/L l-glutamine and Earl salts and supplemented with 10% FBS, 1% sodium pyruvate, and 1% penicillin/streptomycin^42^.

### Cell viability assay

Cytotoxicity was measured using the TACS^®^ MTT cell proliferation assay kit (Trevigen, Gaithersburg, MD, USA). Cells were seeded onto 96-well plates and treated with indicated concentrations of saponin for 48 h. Post treatment, MTT (10 µL per well) was added, and the plates were incubated at 37 °C. Dimethyl sulfoxide (DMSO, 100 µL) was added, and the dark blue formazan product was quantified using a microplate reader at 570 nm (with a 690 nm reference filter) (Molecular Device, Sunnyvale, CA, USA). Relative cell viability (%) is expressed as a percentage relative to non-treated control cells^43^.

### Rg3E treatment

Rg3E powder was provided by Korea Ginseng Corporation. 1 g of Rg3E powder was diluted in 1 ml of PBS by vortexing, keeping it in the water bath (37 °C) for 30 minutes to 1 hour, and then on a shaker for 20–30 minutes. The first filtration was done with 0.8 μm filters, and 0.2 μm filters were used for the second filtration of Rg3E^44,45^.

HESCs from patients with endometriosis and Ishikawa cells were harvested from culture flasks using trypsin/EDTA (0.05%) and plated in 6-well plates (200 mL media) at 37 °C with 95% air and 5% CO_2_ in a humidified environment. HESCs were grown in Ham F12/DMEM (1:1) containing 10% FBS and 1% penicillin/streptomycin. Ishikawa cells were grown in MEM (Invitrogen) containing 2.0 mmol/L l-glutamine and Earl salts supplemented with 10% FBS, 1% sodium pyruvate, and 1% penicillin/streptomycin. At 80% confluency, Rg3E was added at concentrations of 0 µL/mL and 400 µL/mL per the MTT assay results and cultured for 48 h.

### RNA Extraction and Quantitative Real-Time Polymerase Chain Reaction (qRT-PCR)

To assess mRNA concentrations, total RNA was extracted from cultured cells with Qiagen RNeasy isolation kit (Qiagen, Hilden, Germany). The specific method for acquiring mRNA is described in the Supplementary Information. The mRNA levels of each sample were normalized to GAPDH expression^42,43,46–48^.

To assess miRNA expression levels, RNA was extracted from cultured cells using the miRVana RNA Isolation Kit (Ambion by Life Technologies, Carlsbad, CA, USA) per the manufacturer’s specifications and eluted with 30 µL nuclease-free water. Isolated RNA (10 ng) and Taqman MicroRNA Reverse Transcription Kit (Applied Biosystems by Thermo Fisher Scientific, Baltics, UAB, Lithuania) were used. Quantitative real-time PCR for miRNAs was performed using a Taqman^®^ Universal Master Mix II, no UNG (Applied Biosystems, Foster City, CA, USA) with sets for miR-27b-3p and U6 snRNA (Applied Biosystems, Foster City, CA, USA). All real-time PCR reactions were conducted in triplicate with a 7300 real time PCR system, and 40 cycles of amplification were performed. Relative expressions were calculated using the comparative threshold cycle method, and miRNA levels were normalized to U6 levels^42^. Relative miRNA levels were determined using the formula 2^−ΔCt^. Primers used in this experiment are described in the Supplementary Information.

### Migration Assay

The migration assay for transfected cultured cells was carried out using 8-mm pore size polycarbonate membranes (Millipore, Billerica, MA, USA) and 24-well plates. Freshly trypsinized and washed cells were suspended in serum-free medium, and cells (200 mL, 5 × 10^4^ cells/well) were placed in the top chamber of each insert; medium (600 mL) containing 10% FBS was added into the lower chambers. After incubating for 24 h at 37 °C in a 5% CO_2_ humidified incubator, cells were fixed and stained with hematoxylin. Cells in the inner chamber were removed with the use of a cotton swab, and cells attached to the bottom side of the membrane were counted and imaged under an inverted microscope (Olympus Corp., Shinjuku, Tokyo, Japan) at 200 × magnification over ten random fields in each well^42^.

### Collagen Gel Contraction Assay

A sterile solution of bovine Type I Collagen (Cell Biolabs, Inc., San Diego, USA) was prepared per the manufacturer’s instructions. HESCs from the endometrium of the patients with endometriosis treated with Rg3E for 48 h were embedded in collagen gel and cultured three-dimensionally. Briefly, HESCs were suspended in the collagen solution (3.0 × 10^5^ cells/mL). The collagen/cell mixture (2 mL/plate) was dispensed into 35-mm culture plates (Corning, New York, NY, USA); the mixture was polymerized at 37 °C for 1 hour. Immediately after polymerization, 1 mL culture medium was added to each plate. After incubating for 72 h, the collagen gels were photographed and the gel surface area was measured^49,50^.

### miRNA Microarray Analysis

For quality control, RNA purity and integrity were evaluated using an OD 260/280 ratio and analyzed with ND-1000 Spectrophotometer (NanoDrop, Wilmington, USA) and Agilent 2100 Bioanalyzer (Agilent Technologies, Palo Alto, USA). The Affymetrix GeneChip^®^ miRNA array process was conducted per the manufacturer’s protocol. 1 µg RNA samples were labeled with the FlashTag^™^ Biotin RNA Labeling Kit (Genisphere, Hatfield, PA, USA). The labeled RNA was quantified, fractionated and hybridized to the miRNA microarray according to the standard procedures provided by the manufacturer. The labeled RNA was heated to 99 °C for 5 minutes and then to 45 °C for 5 minutes. RNA-array hybridization was performed with agitation at 60 rotations per minute for 16–18 hours at 48 °C on an Affymetrix^®^ 450 Fluidics Station. The chips were washed and stained using a Genechip Fluidics Station 450 (Affymetrix, Santa Clara, California, United States). The chips were then scanned with an Affymetrix GeneChip Scanner 3000 (Affymetrix, Santa Clara, California, United States). Signal values were computed using the Affymetrix^®^ GeneChip^™^ Command Console software. Raw data were extracted automatically in Affymetrix data extraction protocol using the software provided by Affymetrix GeneChip^®^ Command Console^®^ Software (AGCC). The CEL files import, miRNA level RMA + DABG-All analysis and result export were done using Affymetrix^®^ Expression Console^™^ Software. Array data were filtered by probes annotated species.

The comparative analysis between test sample and control sample was carried out using independent t-test and fold change in which the null hypothesis was that no difference exists among groups^51–54^. False discovery rate (FDR) was controlled by adjusting *P*-value using Benjamini-Hochberg algorithm. All statistical tests and visualization of differentially expressed genes were conducted using R statistical language 3.1.2. (www.r-project.org).

### Transfection of miRNA

Cells were cultured to 70–80% confluence after being seeded onto 6-well plates and were transfected with hsa-mir-27b-3p inhibitor, a chemically synthesized double-stranded RNA that inhibits mature endogenous miRNA, or hsa-mir-negative as a control (Ambion by Life Technologies) with the use of Lipofectamine 2000 (Invitrogen) per the manufacturer’s instructions, at a final concentration of 50 nmol/L. The transfected cells were harvested 48 h after transfection^42^.

### Western blot

The protein extracts were prepared using RIPA buffer (Thermo Scientific, Rockford, IL, USA) containing freshly added protease and phosphatase inhibitor cocktail (Thermo Scientific). The concentrations of total cell lysates were measured using a BCA protein assay kit (Thermo Scientific). A total of 20 µg total protein was mixed with 5X sample buffer and heated at 95 °C for 5 min. The samples were loaded onto 8–12% sodium dodecyl sulfate-polyacrylamide gels (SDS-PAGE) and electrotransferred to a polyvinylidene fluoride membrane (Millipore Corporation, Billerica, MA, USA) using a Transblot apparatus (Bio-Rad). The membranes (Millipore Corporation, USA) were blocked using 5% non-fat skim milk in Tris-buffered saline solution (10 mmol/L Tris-HCl (pH 7.4) and 0.5 mol/L NaCl) and adding Tween-20 (0.1% vol/vol).

The blots were probed using primary antibodies: Col-1 (1:1000, Santa Cruz biotechnology), CTGF (1:1000, Santa Cruz biotechnology), Fibronectin (1:1000, Santa Cruz biotechnology), TGF-*β*1 (1:1000, Santa Cruz biotechnology), MMP2 (1:500, Santa Cruz biotechonology), MMP9 (1:500, Santa Cruz biotechnology) and *β*-actin (1:1000, Abcam, Cambridge, UK), followed by horseradish peroxidase conjugated secondary anti-mouse or anti-rabbit antibody (1:2000; Thermo Scientific). The proteins were detected using enhanced chemiluminescence (Santa Cruz Biotechnology, Dallas, TX, USA). The experiment was repeated three times for analysis^55^.

### Mouse Model for Endometriosis

The female mouse model for endometriosis was prepared using 6-week-old C57bl6 female mice as previously described^56^. This study was approved by the institutional committee on animal care and was conducted in accordance with its accepted standards. In brief, the donor mouse was sacrificed under anesthetic overdose and its uterus was obtained. The “Y” shaped uterus was cut in half, and then each uterine horn was dissected so that the endometrium was exposed. The recipient mouse was anesthetized and each uterine horn was transplanted onto each lateral side of the recipient’s peritoneum by vicryl 3-0 sutures. The peritoneum and skin were closed with vicryl 3-0 and staplers, and the postoperative condition of the mice was checked. Rg3E was diluted in PBS as mentioned previously. Rg3E-treated mice were divided into two groups: a high-dose and a low-dose group. There were 10 mice in each group. The high-dose group was given 0.2 mg/g Rg3E and the low-dose group was given 0.1 mg/g Rg3E by oral gavage daily, once a day; the control group was given an equivalent amount of distilled water, also by oral gavage. The Rg3E treatment was started one day after the transplantation date and the total feeding period was 8 weeks.

After 8 weeks of Rg3E treatment, there was no difference in general appearance of the two groups and they were sacrificed for endometriotic tissue retrieval. Endometriotic implants were measured by the length and width of each lesion and their averages were considered for final analysis. The implants were collected, fixed in 10% formalin-acetic acid, and embedded in paraffin for histopathological examination. Paraffin-embedded tissue sections were stained with Masson’s trichrome stain. Masson’s trichrome staining detects collagen fibrils that are deposited in the matrix^29^. To quantify severity of fibrosis in stained tissue sections, staining scores were calculated by multiplying the percentage of positive cells and staining intensity as previously described^29,57^. The mRNA concentrations of invasion and fibrosis markers were also evaluated for mouse model by qRT-PCR as described previously and the primers for mouse study is described in the Supplementary Information.

### Statistical Analysis

Data are presented as means ± SEM. Data from the *in vitro* experiments were assessed by Kolmogorov-Smirnov test or Shapiro-Wilk test to evaluate whether they were normally distributed. Continuous variables were compared using student’s *t*-test or Mann-Whitney U test, when appropriate. The mouse model study data were analyzed by Wilcoxon signed rank test. SPSS v.23.0 and R statistical language v.2.15.0 were used for statistical analyses, and *P* < 0.05 was considered statistically significant.

### Data availability

The datasets generated during and/or analyzed during the current study are available from the corresponding author on reasonable request.

## Electronic supplementary material

Supplementary information
