# Supplementary material for: Ginsenoside Rg3 Decreases Fibrotic and Invasive Nature of Endometriosis by Modulating miRNA-27b: In Vitro and In Vivo Studies
Source: Sci Rep. 2017 Dec 15;7:17670. doi: 10.1038/s41598-017-17956-0 (PMC5732249; doi:10.1038/s41598-017-17956-0)
Supplement: Supplementary file 1 — Supplementary information [file 41598_2017_17956_MOESM1_ESM.doc]

**Ginsenoside Rg3 Decreases Fibrotic and Invasive Nature of Endometriosis by Modulating miRNA-27b: *In Vitro* and *In Vivo* Studies**

Min Kyoung Kim^1,2,3^, Seung Kyun Lee^2,4^, Ji Hyun Park^2,4^, Jae Hoon Lee^1,2^,

Bo Hyon Yun^1,2^, Joo Hyun Park^2,4^, Seok Kyo Seo^1,2^, SiHyun Cho^2,4*^, Young Sik Choi^1,2^

^1^Department of Obstetrics and Gynecology, Severance Hospital, Yonsei University College of Medicine, Seoul 03722, Republic of Korea

^2^Institute of Women’s Life Medical Science, Yonsei University College of Medicine, Seoul 03722, Republic of Korea

^3^Department of Obstetrics and Gynecology, Fertility Center of CHA Gangnam Medical Center, CHA University, Seoul 06125, Republic of Korea

^4^Department of Obstetrics and Gynecology, Gangnam Severance Hospital, Yonsei University College of Medicine, Seoul 06273, Republic of Korea

* [sihyuncho@yuhs.ac](mailto:sihyuncho@yuhs.ac)

**Supplementary Information**

**MTT assay**

**
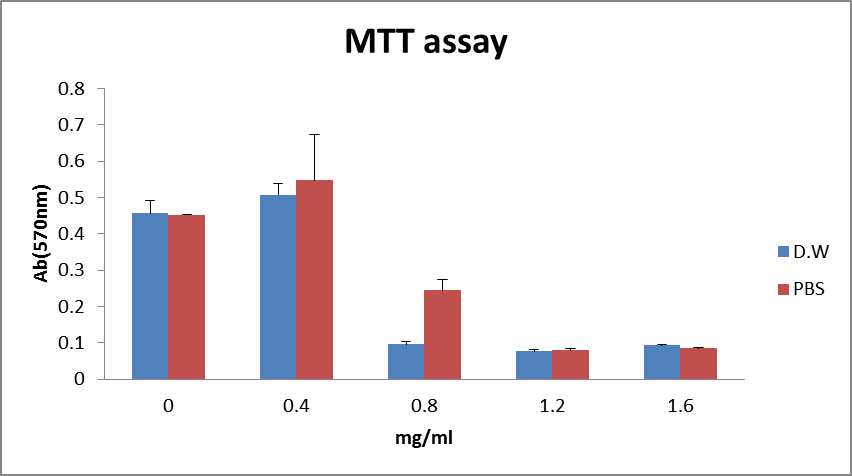
**

MTT assay showed significant decrease in cell viability at 800 µg/mL of Rg3E treatment, and therefore 400 µg/mL of Rg3E was used for in vitro analysis.

**hsa-miR-negative control**

**
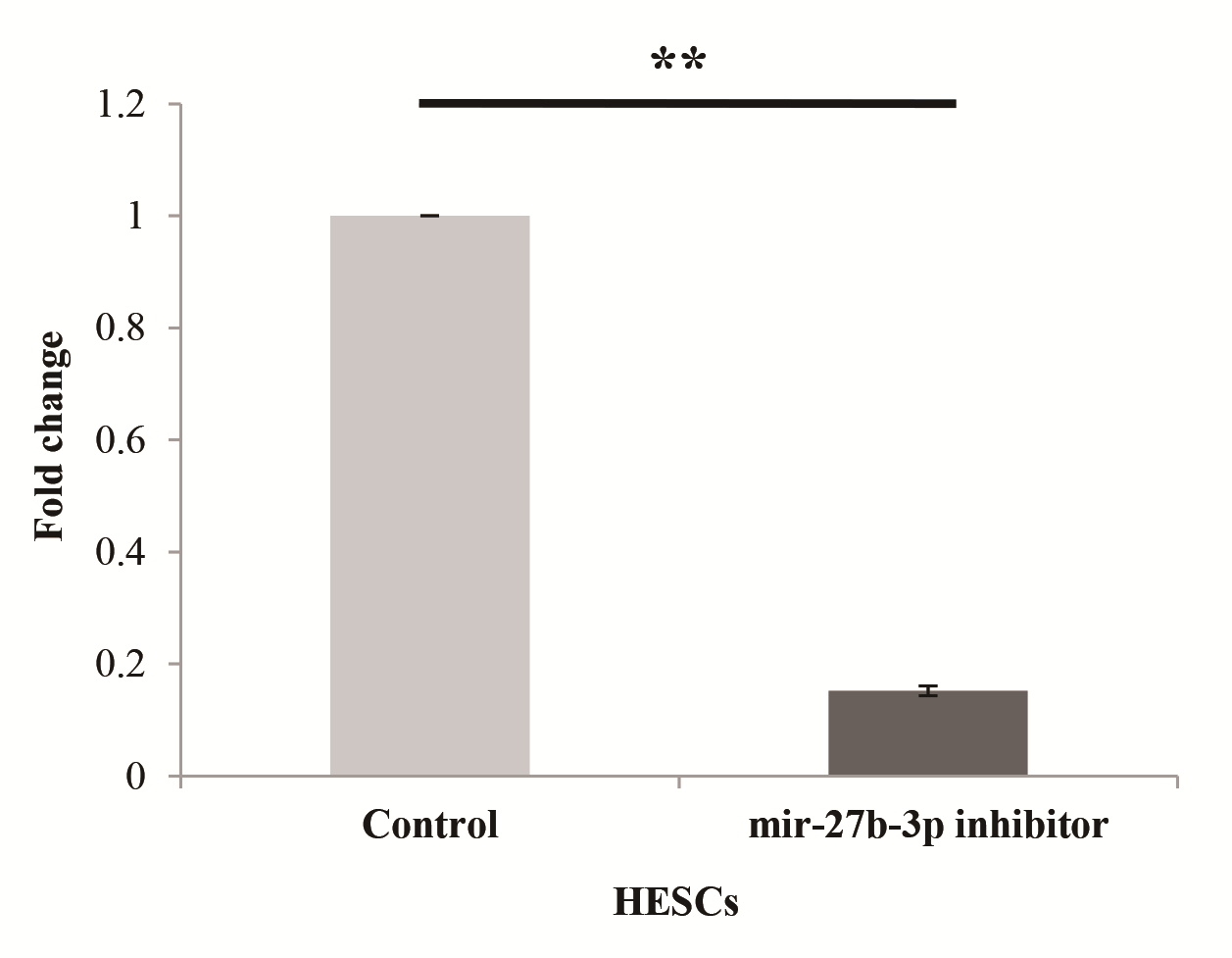
**

Expression of miR-27b-3p was 100- to 200-fold lower after treatment with the has-miR-27b-3p inhibitor than that observed after treatment with the hsa-miR-negative control. (**; *P* < 0.01)

**RNA extraction and quantitative real-time polymerase chain reaction**

All samples were treated with RNase-free DNase (Ambion) to remove the possibility of genomic DNA contamination. RNA samples were analyzed with the use of a Nanodrop ND 2000 spectrophotometer (Thermo Fisher Scientific, Waltham, MA, USA). Using 1 µg of total RNA, cDNA was synthesized with oligo-dT in a Superscript III kit (Invitrogen) with the use of C1000 Thermal Cycler (Bio-Rad, Hercules, CA, USA). The resultant cDNA mixtures were stored at -20°C. Then, using a template of 2µl of synthesized cDNA, Quantitative real-time polymerase chain reaction (PCR) amplification was performed using the 7300 Real Time PCR System (Applied Biosystems, Foster City, CA, USA). Real-time PCR was performed with the use of the Power SYBR Green PCR master mix (Applied Biosystems by Thermo Fisher Scientific, Woolston Warrington, UK). Reaction mixture included cDNA template, forward and reverse primers, ribonuclease free water, and the SYBR Green PCR master mix, for a final reaction volume of 20µL. The thermal cycling conditions were performed by procedures at 95°C for 5 min, followed by 40 cycles of 95°C for 30 sec, 60°C for 30 sec, 72°C for 1 min, and a final extension at 72°C for 5 min. Threshold cycle (Ct) and melting curves were acquired with the use of 7300 software program of the Applied Biosystems. Each reaction was performed in triplicate.

Primers used in this experiment are as following: miR27b-3p forward, TTCACAGTGGCTAAGTTCTGC; U6 forward, 5'-CTCGCTTCGGCAGCACA-3', and reverse, 5'-AACGCTTCACGAATTTGCGT-3'; GAPDH forward, 5'-ACCACAGTCCATGCCATCAC-3', and reverse, 5'-TCCACCACCCTGTTGCTGTA-3; Ki-67 forward, 5'-GAAAGAGTGGCAACCTGCCTTC-3', and reverse, 5'-GCACCAAGTTTTACTACATCTGCC-3'; Caspase-3 forward, 5'-GGAAGCGAATCAATGGACTCTGG-3', and reverse, 5'-GCATCGACATCTGTACCAGACC-3’; CTGF forward, 5'-CATTAAGAAGGGCAAAAAGTGC-3', and reverse, 5'-CACACCCCACAGAACTTAGCC-3'; Col-1 forward, 5’-GAGAGCATGACCGATGGATT-3’, and reverse, 5’-CCTTCTTGAGGTTGCCAGTC-3’; Fibronectin forward, 5'-CCATCGCAAACCGCTGCCAT-3', and reverse, 5'-AACACTTCTCAGCTATGGGCTT-3'; TGF-β1 forward, 5'-TGGAAACCCACAACGAAATC-3', and reverse, 5'-GGGTTCAGGTACCGCTTCTC-3'; MMP2 forward, 5'-ACCGCGACAAGAAGTATGGC-3', and reverse, 5'-CCACTTGCGGTCATCATCGT-3'; MMP9 forward, 5'-CGATGACGAGTTGTGGTCCC-3', and reverse, 5'-TCGTAGTTGGCCGTGGTACT-3'.

**Primers for mouse quantitative real-time polymerase chain reaction**

MMP2 forward, 5'-GCTGTATTCCCGACCGTTGA-3' (20mer), and reverse, 5'-TGGTCCGCGTAAAGTATGGG-3' (20mer); MMP9 forward, 5'-AACATCTGGCACTCCACACC-3' (20mer), and reverse, 5'-GCAGAAGTTCTTTGGCCTGC-3' (20mer); CTGF forward, 5'-TTCCCGAGAAGGGTCAAGCT-3' (20mer), and reverse, 5'-TCCTTGGGCTCGTCACACA-3' (19mer); Col-1 forward, 5'-ATCTCCTGGTGCTGATGGAC-3' (20mer), and reverse, 5'-ACCTTGTTTGCCAGGTTCAC-3' (20mer); Fibronectin forward, 5'-CGAGGTGACAGAGACCACAA-3' (20mer), and reverse, 5'-CTGGAGTCAAGCCAGACACA-3' (20mer); TGF-β1 forward, 5'-GACGTCACTGGAGTTGTACGG-3' (21mer), and reverse, 5'-GCTGAATCGAAAGCCCTGT-3' (19mer).
